# Dry Eye-Related Ocular Surface Assessment in a Pooled Endometriosis/Adenomyosis Cohort: A Real-World Case–Control Study

**DOI:** 10.3390/diagnostics16101524

**Published:** 2026-05-18

**Authors:** Matilde Buzzi, Aurora Tenti, Alberto Carnicci, Carlo Gennaro, Davide Totaro, Maria Volotovskaya, Maria Elisabetta Coccia, Fabrizio Giansanti, Gianni Virgili, Rita Mencucci

**Affiliations:** 1 Eye Clinic, Department of Neuroscience, Psychology, Pharmacology and Child Health (NEUROFARBA), University of Florence, Largo Brambilla 3, 50134 Florence, Italy; 2 Department of Experimental and Clinical Biomedical Sciences “Mario Serio”, University of Firenze, 50134 Florence, Italy

**Keywords:** ocular surface disease, dry eye disease, meibomian gland dysfunction, endometriosis, adenomyosis

## Abstract

**Background/Objectives**: To explore potential dry eye-related ocular surface functional alterations in women at the time of first diagnosis of endometriosis or adenomyosis in a real-world clinical setting. **Methods**: This was a cross-sectional case–control study. Patients were evaluated at the time of initial diagnosis, prior to initiation of any hormonal therapy, to reflect real-world clinical conditions. Participants underwent a standardized ocular surface assessment comprising the Ocular Surface Disease Index (OSDI) questionnaire, Schirmer test, and multimodal TearCheck^®^ analysis, including Non-Invasive Break-Up Time (NIBUT), Tear Film Stability Evaluation (TFSE), Meibography, and Abortive Blinking^®^. **Results**: A total of 71 women were included: 41 with endometriosis or adenomyosis and 30 without known gynecological disease. Patients reported significantly higher OSDI scores than controls (*p* < 0.05). Objective testing demonstrated lower Schirmer values, reduced tear film stability, and more pronounced Meibomian gland dropout in the patient group (all *p* < 0.05). Differences were consistently observed across both subjective and objective parameters. **Conclusions**: Women with endometriosis and/or adenomyosis exhibited significantly altered ocular surface parameters compared with women without known gynecological disease. These findings suggest a possible association between gynecological disease and ocular surface dysfunction. Greater awareness of potential ocular involvement may encourage closer collaboration between gynecology and ophthalmology in the care of affected patients.

## 1. Introduction

Endometriosis is a chronic inflammatory condition characterized by the presence of endometrial tissue outside the uterus, with persistent, invasive, estrogen-dependent growth [1,2]. It typically affects pelvic organs, although extra-pelvic implants have also been reported [1,2,3]. The incidence of endometriosis in women of reproductive age is estimated to be around 10–15%, reaching rates of 30% in patients with infertility and up to 45% in those with chronic pelvic pain [2,4,5]. Adenomyosis is a benign uterine disorder, commonly seen in women of reproductive age, characterized by the presence of endometrial tissue within the myometrium, in a diffuse or focal form [6,7,8]. Although once considered a type of internal endometriosis, it is now recognized as a distinct entity, frequently coexisting with endometriosis. Symptoms also include abnormal uterine bleeding, pelvic pain, and infertility, although about one-third of patients may be asymptomatic. The true prevalence of the disease is difficult to assess due to these asymptomatic forms, but studies based on imaging estimate it at 20–30% [7,9].

Both endometriosis and adenomyosis are characterized by hyperestrogenism, inflammation, and progesterone resistance, three interconnected aspects that contribute to symptoms and infertility in affected patients. Estrogen promotes lesion growth and inflammation, while progesterone resistance hinders anti-inflammatory regulation [10,11]. Both conditions show reduced levels of progesterone receptors (PR-A and PR-B) and altered expression of key enzymes such as aromatase and 17β-HSD2. Therefore, while these hormonal dysregulations are major targets for therapy, one-third of patients respond poorly to progestin treatment, requiring second-line hormonal therapies or surgical interventions [12,13,14].

For what concerns the effects of endometriosis and adenomyosis on the ocular surface, it is known that sex hormones take part in complex mechanisms that variously involve ocular surface structures, such as the cornea, conjunctiva, meibomian and lacrimal glands [15,16]. In patients with endometriosis, elevated estrogen levels have been associated with increased inflammation and Meibomian gland dysfunction, leading to tear film instability and symptoms consistent with dry eye disease [15,17,18]. In various studies, estrogen has been shown to upregulate inflammatory cytokines (IL-1β, IL-6, IL-8) and metalloproteinases (MMP-2, -7, -9) secreted by corneal epithelial cells and lacrimal glands, contributing to ocular surface inflammation [19,20]. Progesterone also affects gene regulation, mainly by reducing protein synthesis in the Meibomian glands, although its effects appear to be more variable [21]. In agreement with the above, sex hormone receptors have been identified in various ocular tissues, indicating a potential pathway for systemic hormonal disorders to affect the eye. Hormonal fluctuations, especially in estrogen and progesterone levels, may therefore play a significant role in the pathogenesis of DED in conditions like endometriosis and adenomyosis [18,20,22,23].

In recent years, dry eye disease (DED) has been increasingly recognized as a multifactorial disorder of the ocular surface characterized by tear film instability, hyperosmolarity, inflammation, and neurosensory abnormalities. Importantly, endometriosis is now considered a chronic systemic inflammatory disease rather than a condition confined to the pelvic organs [3]. Circulating inflammatory mediators, altered immune responses, and persistent hormonal imbalance may potentially influence distant tissues, including the ocular surface. Despite a plausible biological link, evidence on ocular surface involvement in endometriosis and adenomyosis is limited, and the extent to which these conditions contribute to clinically significant ocular surface changes remains unclear.

A better characterization of this potential association may contribute to improved recognition of ocular symptoms in affected women and provide a foundation for future investigations.

The aim of this study was to investigate the potential impact of endometriosis and adenomyosis on the ocular surface, with regard to both functional and structural alterations. The novelty of this study lies in the real-world, multimodal assessment of ocular surface parameters in women with endometriosis and adenomyosis evaluated at the time of first diagnosis, before initiation of hormonal therapy.

## 2. Materials and Methods

A cross-sectional observational case–control study was conducted at the Eye Clinic of the Azienda Ospedaliero-Universitaria Careggi in Florence, Italy. The study was conducted in accordance with the tenets of the Declaration of Helsinki and was approved by the local Institutional Ethics committee. Verbal and written informed consent were obtained from all participants prior to enrollment.

Patients were recruited from August 2024 to April 2025 by the Obstetrics and Gynecology Department of the Careggi University Hospital of Florence and constituted the case group. Diagnosis of endometriosis or adenomyosis was established by gynecological specialists based on routine clinical practice, integrating clinical evaluation and imaging findings in accordance with internationally accepted diagnostic criteria. Transvaginal ultrasound (TVUS) represented the initial diagnostic assessment in all patients included in the case group. In selected cases, magnetic resonance imaging (MRI) and/or prior laparoscopic or histopathological findings were additionally available and used as confirmatory information when clinically indicated. Because of the real-world design of the study, several patients underwent more than one complementary diagnostic modality during routine clinical evaluation. Only patients evaluated at the time of their initial specialist diagnosis at our institution, prior to initiation of systemic hormonal treatment, were included. Conversely, the control group was composed of patients recruited during routine visits in the same period at the Ophthalmology Clinic of the same facility.

Inclusion criteria were age > 18 years; ability to provide informed consent; confirmed diagnosis of endometriosis or adenomyosis (case group); and absence of these conditions (control group). In the control group, the absence of endometriosis or adenomyosis was verified through medical history review and absence of prior gynecologic diagnosis. No additional gynecological imaging was performed for study purposes. Exclusion criteria for both groups included: history of intraocular or refractive surgery; ocular surface or adnexal disease (e.g., Sjögren’s syndrome, conjunctivitis, keratitis); use of topical ocular lubricants or any other ocular medications; contact lens wear; and systemic conditions such as diabetes mellitus, rheumatologic, or other autoimmune diseases, as well as patients undergoing short-term or chronic pharmacological treatments for these conditions, including antidepressants and antihistamines. None of the participants in either the endometriosis/adenomyosis group or the control group had received systemic hormonal therapy, including oral contraceptives or other hormone-based treatments, during the 12 months prior to enrollment.

### 2.1. Clinical Examination and Schirmer Test

All patients underwent an initial evaluation with slit-lamp biomicroscopy to exclude potentially confounding ocular conditions. Best-corrected visual acuity was not included among the study outcomes because the investigation was specifically focused on dry eye-related ocular surface parameters rather than visual function. The examination was performed in a standardized sequence to avoid reflex tearing and measurement interference, assessing the ocular surface structures and specifically excluding any sign of conjunctivitis or keratoconjunctivitis.

For a quantitative assessment of tear production, the Schirmer I test was performed without anesthesia, with patients keeping their eyes closed for 5 min. Strips of millimeter blotting paper measuring 5 mm × 30 mm, labeled with the letters “L” and “R” to distinguish the left and right eye, respectively, were used to perform the test. Each strip, properly folded at 90°, was inserted into the inferior conjunctival fornix, between the palpebral and bulbar conjunctiva. Patients were instructed to keep their eyes gently closed for five minutes; afterwards, the strips were removed, and the length of the wet portion was measured in millimeters [24].

### 2.2. TearCheck Instrumental Examination

TearCheck^®^ (ESW Vision, Houdan, France) is a high-tech diagnostic method designed to noninvasively evaluate ocular surface health [25]. For the purposes of this study, the following TearCheck^®^ examinations were performed: NIBUT (Non-invasive Breakup Time), TFSE (Tear Film Stability Evaluation), Meibography and Abortive Blinking. All TearCheck^®^ measurements were performed by the same experienced examiner to reduce inter-operator variability. All examinations were performed under standardized room conditions, with controlled ambient illumination and a fixed examination sequence for all participants. Subjects were allowed to adapt to the examination room before testing, and all measurements were obtained before contact procedures in order to minimize reflex tearing and improve repeatability. NIBUT is a noninvasive test used to measure the tear film break-up time. It is determined using the Placido disk, which projects concentric rings onto the cornea. Then, the time between a complete blink and the first distortion of the reflected image on the corneal surface is measured [25]. TFSE assesses tear film stability by analyzing micro-deformations on the tear surface with a ten-second recording for each eye. Meibography is a noninvasive infrared imaging technique used to visualize the morphology of the Meibomian glands and detect structural alterations. TearCheck^®^ enables quantification of Meibomian gland loss as a percentage and provides both 2D images and 3D reconstructions. Finally, the Abortive Blinking function^®^ allows all eyelid blinks to be recorded in a 60-s interval, providing automatic quantification of both the total number of blinks and the percentage of ineffective blinks. Grading scales of the abovementioned parameters are reported in Table 1.

### 2.3. Ocular Surface Disease Index (OSDI) Questionnaire

The Ocular Surface Disease Index (OSDI) is a validated instrument developed by the Outcomes Research Group at Allergan Inc. in 1995 to quantify symptoms of dry eye disease (DED) and their impact on vision-related functioning [26]. In this study, the Italian version of the OSDI was administered to all participants to assess subjective symptom burden [27]. The questionnaire comprises three subscales: ocular symptoms (e.g., photophobia, foreign body sensation), functional limitations (e.g., reading, night driving), and environmental triggers (e.g., wind, air conditioning). Participants rated symptom frequency over the previous week using a 5-point Likert scale (0 = none of the time to 4 = all of the time). Total scores were computed and normalized to a 0–100 scale, with higher scores indicating greater symptom severity.

### 2.4. Statistical Analysis

Statistical analysis was performed using IBM SPSS Statistics for Windows (version 30.0, IBM Corp., Armonk, NY, USA) and Stata software (version 19.0, StataCorp, College Station, TX, USA). Descriptive statistics were computed for all variables. Continuous variables are presented as mean ± standard deviation (SD) with corresponding 95% confidence intervals (CI), while categorical variables are reported as frequencies and percentages. Linear regression was employed to compare the values of continuous variables between groups, adjusting for intra-subject correlation between both eyes by clustering on each individual and applying a robust variance estimator (Huber-White). A sensitivity analysis excluding patients with isolated adenomyosis was additionally performed to assess the robustness of the findings. In addition, age-adjusted regression analyses were performed to account for potential confounding related to age differences between groups. A *p*-value of less than 0.05 was considered statistically significant.

## 3. Results

Seventy-one female patients were enrolled in this study, for a total of 142 eyes. The case group included 41 patients (82 eyes), with a mean age of 33.68 ± 8.58 years. The control group consisted of 30 patients (60 eyes), with a mean age of 33.83 ± 16.39 years. Table 2 presents patients’ demographic and clinical characteristics. No statistically significant difference in age was observed between the case and control groups (*p* > 0.05) and the 95% confidence intervals showed substantial overlap between groups, supporting comparable age distribution at baseline. Table 3 reports the results of the OSDI questionnaire, Schirmer test, and TearCheck^®^ examinations in both groups. Sensitivity analysis excluding patients with isolated adenomyosis confirmed similar effect sizes and statistical significance across all principal ocular surface parameters (Table S1) and age-adjusted regression analyses confirmed the direction and statistical significance of all primary outcomes (Table S2).

**Figure 1:**
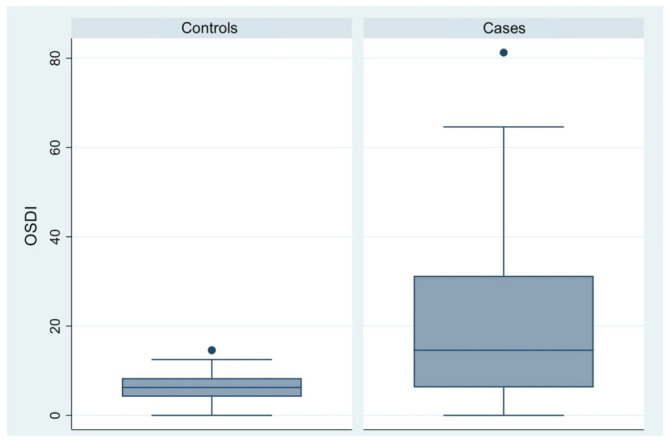
Box plot comparison of Ocular Surface Disease Index (OSDI) scores between control participants and patients with endometriosis or adenomyosis.

The mean OSDI questionnaire score was significantly higher in the case group (95% CI, 16.86–25.71) than in the control group (95% CI, 5.43–7.63), with a mean difference of +14.76 points. The box plot shows greater spread in the case group, suggesting higher variability in symptom severity (Table 3, Figure 1).

**Figure 2:**
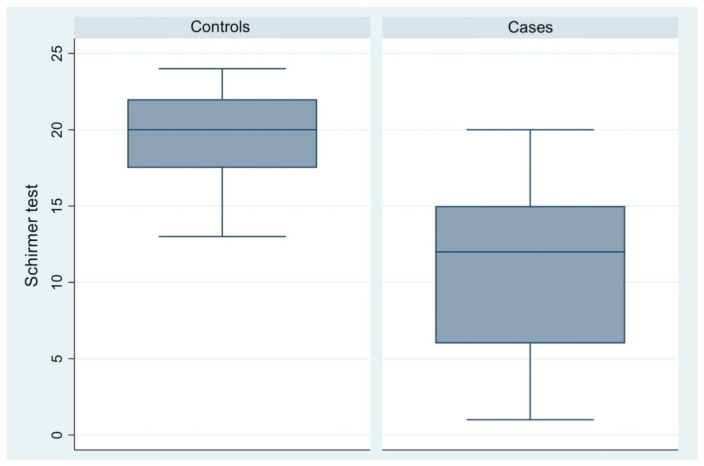
Box plot comparison of Schirmer test scores between control participants and patients with endometriosis or adenomyosis.

A mean difference of 8.77 mm was observed in Schirmer test values between the control group (95% CI, 18.51–20.02) and the case group (95% CI, 9.42–11.58) (Table 3, Figure 2).

**Figure 3:**
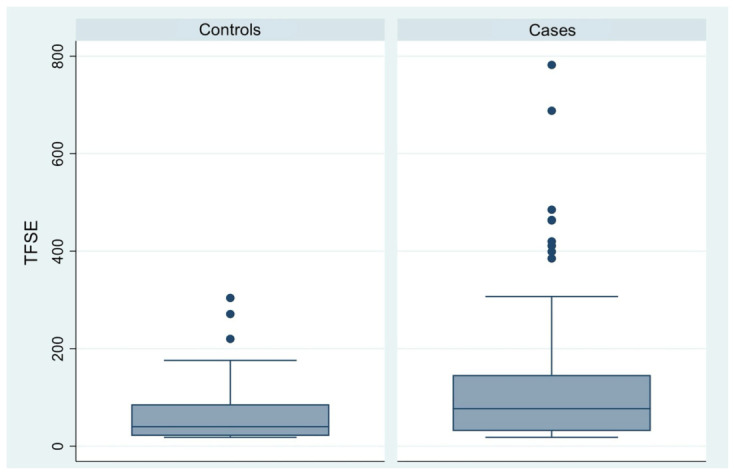
Box plot comparison of Tear Film Stability Evaluation (TFSE) scores between control participants and patients with endometriosis or adenomyosis.

Mean TFSE values were higher in the case group (95% CI, 98.47–165.99) compared to controls (95% CI, 49.12–81.58), with a mean difference of approximately 66.88. Despite noticeable variability and the presence of outliers in both groups, the difference remained statistically significant (Table 3, Figure 3).

**Figure 4:**
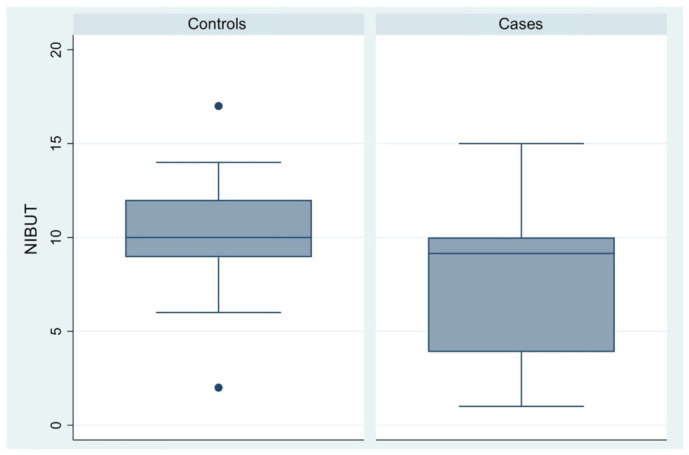
Box plot comparison of Non-invasive Break-up Time (NIBUT) scores between control participants and patients with endometriosis or adenomyosis.

Figure 4 illustrates a significant between-group difference in NIBUT values, with a mean disparity of 2.90 s. Patients in the case group (95% CI, 6.71–8.30) had shorter tear break-up times compared to control group (95% CI, 9.75–11.05), indicating lower tear film stability. Greater variability was again observed in the case group, possibly reflecting the differences in symptom severity (Table 3, Figure 4).

**Figure 5:**
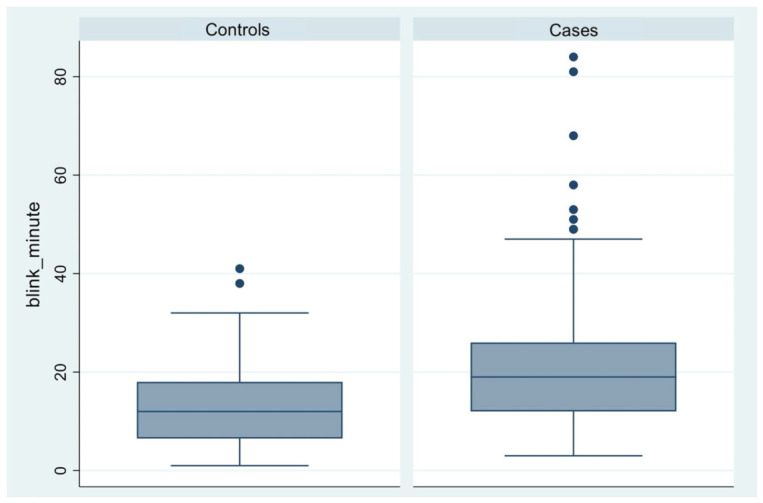
Box plot comparison of Blink rate scores between control participants and patients with endometriosis or adenomyosis.

Patients in the case group (95% CI, 18.93–26.15) exhibited a significantly higher average blink rate compared to the control group rate (95% CI, 11.33–15.84) with a mean difference of 8.96 blinks/min. (Table 3, Figure 5).

**Figure 6:**
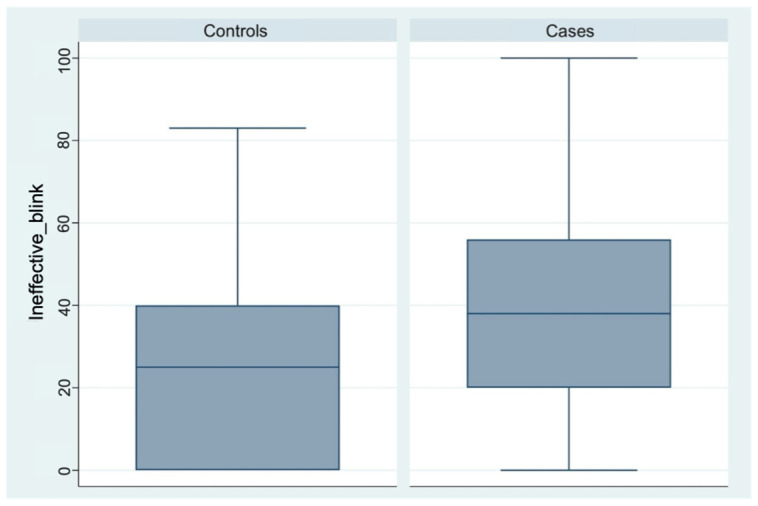
Box plot comparison of Ineffective blink scores between control participants and patients with endometriosis or adenomyosis.

The proportion of ineffective blinks was higher in the case group (95% CI, 34–45) than in controls (95% CI, 18–30), with a statistically significant difference (Table 3, Figure 6).

**Figure 7:**
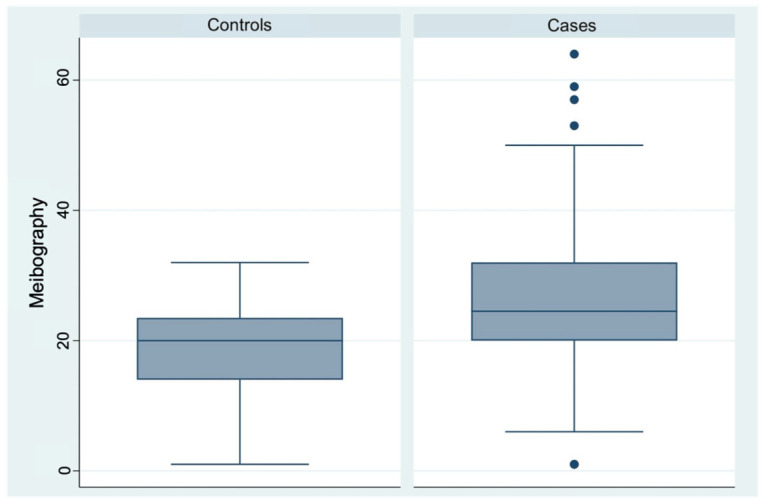
Box plot comparison of Meibography scores between control participants and patients with endometriosis or adenomyosis.

Meibography analysis showed a 28% loss of Meibomian glands in the case group (95% CI, 25–30), compared to 19% in the control group (95% CI, 17–21) and this difference of 9 percentage points was statistically significant (Table 3, Figure 7).

## 4. Discussion

The purpose of the study was to evaluate whether gynecological conditions such as endometriosis and adenomyosis could affect the ocular surface, leading to both functional and structural alterations. Our findings highlight a significant alteration of the ocular surface in patients with endometriosis or adenomyosis compared to age- and sex-matched controls without known gynecological disease. Specifically, the OSDI questionnaire, tear production assessed by the Schirmer Test, and data obtained using TearCheck^®^ device (NIBUT, TFSE, Meibography, and Abortive Blinking^®^) were all negatively affected in the case group, with statistically significant differences compared to the control group. These results suggest that the gynecological conditions examined may influence ocular surface pathophysiological mechanisms, inducing both subjective and objective changes, likely through a possible systemic involvement of the disease. While limited by its cross-sectional design, this study provides a real-world snapshot of ocular surface status at the earliest stage of disease diagnosis, generating hypotheses for future prospective investigations.

There are few studies in the literature that have specifically investigated the correlation between these gynecological conditions and the ocular surface. However, a 2020 study by Turan et al. found that patients with endometriosis had significantly lower mean values in both the Schirmer and TBUT tests compared to controls [18]. Moreover, the mean OSDI scores and alterations detected by conjunctival impression cytology (CIC) were significantly higher. Specifically, CIC revealed a significant decrease in goblet cell numbers and an increase in morphological alterations of the conjunctival epithelium, such as squamous metaplasia. This study demonstrated that women with endometriosis experience greater tear film instability and a higher prevalence of dry eye [18].

From a pathophysiological perspective, several factors may help explain the correlation between endometriosis or adenomyosis and dry eye disease. Both conditions are characterized by hyperestrogenism, documented through elevated circulating estrogen levels, altered progesterone response, and chronic inflammation [10,17]. At the ocular level, various studies have demonstrated the presence of estrogen and progesterone receptors, as well as altered secretion of pro-inflammatory cytokines and metalloproteinases, regulated in response to hormonal imbalances [19,20,22]. Moreover, estrogen is implicated in the regulation of gene expression in Meibomian glands, influencing protein synthesis and lipid metabolism, and exerts a pro-inflammatory effect that inhibits glandular secretion [18,21].

Physiological hormonal fluctuations during the menstrual cycle have been shown to induce mild variations in tear production and stability in healthy women [19,20]. Because the menstrual phase was not controlled and circulating hormone levels were not measured in our study, we cannot exclude that part of the variability observed in ocular surface parameters may reflect physiological hormonal fluctuations. Nevertheless, the consistent differences observed between patients and controls across both subjective and objective measures suggest a potential association between endometriosis/adenomyosis and ocular surface dysfunction that warrants further investigation.

As for the effects of pharmacological therapy used in the treatment of endometriosis and adenomyosis, clinical study results are conflicting. Nonetheless, it has emerged that in women of reproductive age, the use of oral contraceptives does not seem to significantly affect the ocular surface or Meibomian gland secretion. However, these results may be influenced by the type of molecule administered, the dosage, treatment duration, and the individual response [13,14,19,21]. It should be emphasized that none of the participants included in our study had received systemic hormonal therapy, including progestin-based treatments or oral contraceptives, during the 12 months preceding enrollment. Therefore, the ocular surface alterations observed in our cohort cannot be attributed to active pharmacological hormonal modulation.

Notably, our study demonstrated a multifaceted pattern of ocular surface impairment in patients with endometriosis or adenomyosis, revealing both functional and structural alterations across all tested parameters. In addition to significantly higher OSDI scores, patients in the case group exhibited markedly reduced tear production (Schirmer test), lower tear film stability (NIBUT), and significantly impaired lipid layer dynamics (TFSE and Meibography). These findings were accompanied by pronounced abnormalities in blink patterns, including increased blink rate and a significantly higher proportion of ineffective blinks. These alterations suggest that the ocular surface in affected patients is subject to a combination of evaporative and aqueous-deficient dry eye mechanisms. The presence of a hyperblinking phenotype alongside a greater rate of incomplete blinking likely reflects a compensatory response to tear film instability, which paradoxically may worsen evaporative loss by disrupting lipid layer spread. Moreover, the increased Meibomian gland dropout observed in the case group supports the hypothesis of hormonally or inflammation-mediated glandular dysfunction as a key driver of these changes. These findings suggest a possible association between endometriosis/adenomyosis and ocular surface alterations, potentially mediated by hormonal, inflammatory, and neurosensory mechanisms. Because these techniques provide quantitative biological measurements, careful standardization of acquisition and awareness of physiological variability remain essential for correct interpretation of ocular surface data [28].

The main limitations of this study are the relatively small sample size and the absence of long-term follow-up, which precludes evaluation of the progression of ocular alterations over time. In particular, the adenomyosis subgroup was limited in size, preventing reliable subgroup analyses and limiting the ability to draw condition-specific conclusions for adenomyosis. In addition, although transvaginal ultrasound (TVUS) represented the initial diagnostic assessment in all patients, several subjects underwent complementary investigations, including magnetic resonance imaging (MRI) and, in selected cases, prior laparoscopic or histopathological confirmation, according to routine clinical practice. Because of the real-world clinical design of the study and the multimodal diagnostic work-up adopted in many patients, it was not possible to consistently classify subjects according to a single exclusive diagnostic modality, potentially introducing diagnostic heterogeneity. Moreover, because control subjects were identified on the basis of medical history alone, the presence of undiagnosed endometriosis or adenomyosis in asymptomatic individuals cannot be completely excluded. Furthermore, none of the enrolled patients had received hormonal therapy in the 12 months preceding enrollment, which may limit the representativeness of the cohort, as many patients with endometriosis or adenomyosis are commonly managed with hormonal treatments. In addition, the absence of corneal and conjunctival staining prevented a direct clinical assessment of ocular surface epithelial involvement. Another limitation is the absence of menstrual cycle phase standardization and the lack of serum hormonal measurements at the time of ocular examination. Because ocular surface homeostasis may vary according to physiological fluctuations in estrogen and progesterone, these missing data limit the interpretation of the observed findings and prevent a more precise understanding of the relationship between gynecological disease and ocular surface alterations. However, this reflects the real-world design of the study, in which patients were evaluated at the time of diagnosis without prior hormonal assessment. While this may have introduced variability, it does not invalidate the observed between-group differences, which were consistent across multiple independent parameters. Future prospective studies incorporating menstrual cycle stratification and systemic hormonal profiling will be essential to determine whether the observed ocular surface changes are related to disease-specific mechanisms or to physiological hormonal variability. Potential research should focus on identifying patient subgroups at higher risk for ocular involvement, considering factors such as disease duration and type of hormonal therapy. Larger studies, including assessments of estrogen levels in blood and tear samples, may help clarify the relationship between endometriosis and dry eye disease.

## 5. Conclusions

In conclusion, this study shows that women with endometriosis and adenomyosis exhibit significant ocular surface alterations, with consistent impairment in tear secretion, tear film stability, and symptom burden compared with controls without known gynecological disease. These findings suggest that, although these gynecological disorders primarily affect non-ocular sites, their systemic hormonal and inflammatory effects may extend to the ocular surface and compromise its homeostasis. A key aspect of the present study is its real-world design, as patients were evaluated at the time of first diagnosis, without stratification for menstrual cycle phase or circulating hormone levels. While this approach may introduce physiological variability, it reflects routine clinical practice and provides an unselected overview of ocular surface status in this patient population. These findings should be interpreted as representative of real-world clinical conditions at diagnosis, rather than hormonally controlled experimental settings. Future studies may clarify whether ophthalmologic screening could be clinically useful and whether a multidisciplinary approach may help to optimize diagnosis, management, and overall care in this patient population.

## Figures and Tables

**Table 1:** Grading scale of Dry Eye Disease severity, based on TearCheck^®^ diagnostic parameters.

Examination	Unit	Normal	Mild	Moderate	Severe
NIBUT	Time (s)	>10.0	10.0–6.0	5.9–2.0	<2.0
TFSE	Score	<100	100–250	251–500	>500
Meibography	Loss %	<20	20–35	36–50	>50
Abortive Blinking					
Blinks per minute	Blink/min	<20	20–25	26–32	>32
Ineffective blinks	%/min	<15	15–25	26–50	>50

**Table 2:** Baseline demographic and clinical characteristics of study participants in case and control groups.

	Cases	Controls
Patients (eyes)	41 (82)	30 (60)
Age (years, mean ± SD)	33.68 ± 8.58 (95% CI, 31.80–35.57)	33.83 ± 16.39 (95% CI, 29.60–38.07)
Pathology		
Adenomyosis *n* (%)	7 (17%)	-
Endometriosis *n* (%)	34 (83%)	-

**Table 3:** Comparative analysis of clinical and imaging parameters between case and control groups. Abbreviations: OSDI, Ocular Surface Disease Index; TFSE, Tear Film Stability Evaluation; NIBUT, Non-Invasive Break-up Time. All parameters are reported as mean ± standard deviation (SD), along with 95% confidence intervals (CI).

Parameters	Cases	Controls	*p*-Value
OSDI (score)	21.29 ± 20.13 (95% CI, 16.86–25.71)	6.53 ± 4.26 (95% CI, 5.43–7.63)	<0.001
Schirmer test (mm)	10.50 ± 4.91 (95% CI, 9.42–11.58)	19.27 ± 2.92 (95% CI, 18.51–20.02)	<0.001
TFSE	132.23 ± 153.67 (95% CI, 98.47–165.99)	65.35 ± 62.84 (95% CI, 49.12–81.58)	0.002
NIBUT (s)	7.50 ± 3.64 (95% CI, 6.71–8.30)	10.40 ± 2.52 (95% CI, 9.75–11.05)	<0.001
Blinks per minute (blink/min)	22.54 ± 16.43 (95% CI, 18.93–26.15)	13.58 ± 8.73 (95% CI, 11.33–15.84)	<0.001
Ineffective blinks (%/min)	39 ± 25 (95% CI, 34–45)	24 ± 22 (95% CI, 18–30)	<0.001
Meibography (%)	28 ± 12 (95% CI, 25–30)	19 ± 7 (95% CI, 17–21)	<0.001

## Data Availability

The data presented in this study are available on request from the corresponding author due to privacy and ethical restrictions.

## Supplementary Materials

The following supporting information can be downloaded at: https://www.mdpi.com/article/10.3390/diagnostics16101524/s1, Table S1: Sensitivity analysis excluding patients with isolated adenomyosis, showing comparative ocular surface outcomes between the endometriosis subgroup and controls without known gynecological disease; Table S2: Age-adjusted regression analyses for primary ocular surface parameters.
