# Supplementary material for: Dry Eye-Related Ocular Surface Assessment in a Pooled Endometriosis/Adenomyosis Cohort: A Real-World Case–Control Study
Source: Diagnostics (Basel). 2026 May 18;16(10):1524. doi: 10.3390/diagnostics16101524 (PMC13205344; doi:10.3390/diagnostics16101524)
Supplement: Supplementary file 1 [file diagnostics-16-01524-s001.zip › diagnostics-4249727-supplementary.pdf]

Table S1. Sensitivity analysis excluding patients with isolated adenomyosis, showing comparative ocular surface outcomes between the endometriosis subgroup and controls without known gynecological disease.

Parameters	Endometriosis only	Controls	p-value
OSDI *(score)*	21.44 ± 21.58 (95% CI, 16.31-26.57)	6.53± 4.26 (95% CI, 5.43-7.63)	<0.001
Schirmer test *(mm)*	10.59 ± 5.08 (95% CI, 9.38-11.80)	19.27 ± 2.92 (95% CI, 18.51-20.02)	<0.001
TFSE	134.53 ± 156.72 (95% CI, 97.28-171.78)	65.35 ± 62.84 (95% CI, 49.12-81.58)	0.002
NIBUT *(sec)*	7.27 ± 3.55 (95% CI, 6.42-8.11)	10.40± 2.52 (95% CI, 9.75-11.05)	<0.001
Blinks per minute *(blink/min)*	23.53± 16.56 (95% CI, 19.60-27.47)	13.58± 8.73 (95% CI, 11.33-15.84)	<0.001
Ineffective blinks *(%/min)*	41± 24 (95% CI, 35-47)	24± 22 (95% CI, 18-30)	<0.001
Meibography *(%)*	28±13 (95% CI, 25-31)	19±7 (95% CI, 17-21)	<0.001

Table S2. Age-adjusted regression analyses for primary ocular surface parameters.

Parameters	Age-adjusted p-value
OSDI	<0.001
Schirmer test	<0.001
TFSE	0.001
NIBUT	<0.001
Blinks per minute	<0.001
Ineffective blinks	<0.001
Meibography	<0.001
